# The *no correlation* argument: can the morality of conscientious objection be empirically supported? the Italian case

**DOI:** 10.1186/s12910-017-0221-x

**Published:** 2017-11-21

**Authors:** Marco Bo, Carla Maria Zotti, Lorena Charrier

**Affiliations:** 1 Research Group in Bioethics, University of Turin, Turin, Italy; 2 Consulta di Bioetica onlus, Turin, Italy; 3 Department of Public Health and Pediatrics, University of Turin, Via Santena 5 bis, 10126 Torino, Italy

**Keywords:** Conscientious objection, Voluntary abortion, Waiting times, Correlation, Moral principles, Moral integrity, Right

## Abstract

**Background:**

The legitimacy of conscientious objection to abortion continues to fuel heated debate in Italy. In two recent decisions, the European Committee for Social Rights underlined that conscientious objection places safe, legal, and accessible care and services out of reach for most Italian women and that the measures that Italy has adopted to guarantee free access to abortion services are inadequate. Nevertheless, the Ministry of Health states that current Italian legislation, if appropriately applied, accommodates both the right to conscientious objection and the right to voluntary abortion.

**Main body:**

One empirical argument used to demonstrate that conscientious objection does not create barriers to abortion is the “no correlation” argument, which the Italian Committee for Bioethics employed to demonstrate that no association exists between conscientious objection and waiting times for voluntary abortion in Italy and to support the weak form of conventional comprise adopted by the Italian legislation to balance the conflict between women’ autonomy and healthcare professionals’ moral integrity. Conversely, we showed how the “no correlation” argument fails to demonstrate the absence of a relationship between the number of conscientious objectors and waiting times for voluntary abortion, and that the limitations of the “no correlation” argument itself demonstrate how it is still difficult to describe the real effect of conscientious objection on the access to abortion services and to evaluate the suitability of conventional compromise to effectively balance conflicting moral principles.

**Conclusion:**

Further studies are needed to better describe the relationship between conscientious objection and waiting times for voluntary abortion. If new evidence would show that the increasing proportion of objectors does undermine the efficacy of the Italian law and the right of a woman to freely obtain a voluntary abortion, new ways will need to be found to address the conflict between moral principles and restrict the protection accorded to the principle of moral integrity. This would inevitably imply the need to constrain and to redefine the terms and conditions for claiming conscientious objection.

## Background

The legitimacy of conscientious objection to abortion continues to fuel heated public debate in Italy. Media reports of barriers to abortion created by the lack of hospital staff willing to perform termination of pregnancy have galvanized opinion on both sides of the issue [1, 2]. The Italian Ministry of Health has countered these claims by arguing that current Italian legislation – when appropriately applied – accommodates the right to conscientious objection and the right to voluntary termination of pregnancy.

In response, stakeholders and a trade union filed collective claims with the European Committee for Social Rights (ECSR). In two recent decisions, the Committee stated that Italy violated art. 11§1 (right to health) and art. E (right to no discrimination) of the European Convention of Social Rights. In particular, the ECSR underlined that conscientious objection places safe, legal, and accessible care and services out of reach for most Italian women and that the measures that Italy has adopted remain inadequate [3, 4].

To demonstrate that the right to exercise conscientious objection does not deny the right to obtain abortion, the Government principally resorted to empirical data from the Italian healthcare information system on voluntary abortion. One of the empirical arguments rallied to demonstrate that conscientious objection does not create a barrier to access to abortion has been firstly proposed by the Italian Committee for Bioethics (CNB) in its opinion statement on conscientious objection in healthcare and qualified as the “no correlation argument”. The no correlation argument works as follows: by comparing from two non-consecutive years the proportion of objectors and the proportion of women who obtain abortion promptly (within 14 days from the request) or later (between 22 and 28 days), data show different if not opposing “trends” at the regional level (in some regions waiting time decreases as the proportion of objectors increases, while in others waiting time increases as the proportion of objectors decreases). Based on these results, the no correlation argument concludes that no relationship exists between conscientious objection and waiting times for voluntary abortion in Italy, and that waiting time is a consequence of the way regional healthcare systems organize the delivery of abortion services. Thus, conscientious objection per se does not impair women’s right to abortion [5].

This paper aims to show that the no correlation argument fails to demonstrate the absence of correlation between conscientious objection and waiting times for voluntary abortion in Italy and that the argument cannot exclude an association between the two phenomena. Moreover, the paper will show that the limitations of the no correlation argument underline that it is still difficult to understand the effect of conscientious objection on voluntary abortion in Italy and that using an empirical argument to sustain the morality of conscientious objection raises a series of questions about which instruments should be used to describe facts and to empirically demonstrate or exclude causal effects.

## Main text

### Conscientious objection to voluntary abortion in Italy: Legislation and balance between moral principles

Under Italian law 194/78 on voluntary abortion, a woman may freely and anonymously obtain a voluntary abortion in a public hospital. In order to obtain abortion, a woman must submit a written request to an authorized physician. After having verified that the legal requirements for a voluntary abortion have been met, the physician must issue a medical certificate. But the law imposes a 7-day waiting period before the woman can go to a hospital to obtain termination of pregnancy.

Healthcare providers can claim conscientious objection to performing an abortion. The refusal to perform abortions is definitive, and healthcare providers can claim conscientious objection by written statement at any time in their career. Objectors are under no obligation to explain or justify the reasons for their objection or to perform other duties to compensate for their decision not to perform abortions. The Italian abortion law mandates the regional healthcare systems to organize the delivery of abortion services through staff mobility and differentiated recruitment of non-objecting providers. Further, the regional healthcare systems must collect annual data about the number of voluntary abortions performed and the number of conscientious objectors on duty at facilities. Finally, the Ministry of Health sends the Parliament an annual national report on the application of the law.

Theoretically, the Italian law on voluntary abortion seeks to strike a compromise between the principle of reproductive autonomy – which states that an individual has the right to decide whether and how to reproduce – and the right to individual moral integrity – which states a person cannot be forced to act against his/her own conscience. According to Italian law, prima facie both principles should assume equal value, so that they must receive the same degree of legal protection: women can obtain an abortion and healthcare providers can opt out of performing it.

Nevertheless, any healthcare provider can unquestionably refuse to perform an abortion, independent of the number of objectors who are already on duty. Thus, the proportion of objectors may theoretically increase and raise the total number of healthcare providers available in a geographic area. In such a scenario, the State has no way of guaranteeing the right to abortion, because there could be no more professionals willing to perform the procedure.

It is evident that although prima facie the law was intended to ensure equal value to both principles, it has the inherent the risk of conferring an absolute value to the principle of moral integrity, thus overriding the principle of reproductive autonomy which has de facto a relative value.

Beyond such extreme situations, it is rational to recognize that it is difficult – if not impossible – to ensure the adequate provision of abortion services when the proportion of conscientious objectors is as high as it now is in some Italian regions (80.7% in Abbruzzi and Latium, 93.3% in Molise). Under these conditions and given the low number of non-objectors, it is unlikely that regional healthcare services and local health trusts can appropriately guarantee this right by implementing management strategies such as conflating abortion services, assigning non-objecting physicians or resorting to non-objector staff mobility to cover them. It is not by coincidence that many women have reported they faced serious difficulties to obtain an abortion within the first trimester of pregnancy or had to travel to another province, region or country to obtain an abortion.

In this context, defense of the current legal right to conscientious objection rests on the ability to demonstrate that access to abortion services is ensured even when a healthcare provider’s right to exercise conscientious objection is unquestionable.

### The *no correlation* argument

The no correlation argument, as first expressed by the CNB, was developed according to the following lines: the proportion of objectors and the proportion of women who obtained an abortion promptly (within 14 days of submission of their request) or later (between 22 and 28 days) were compared by region and nationally for two non-consecutive years (2006 and 2009) [5]. The analysis showed that when 2006 and 2009 were directly compared, the proportion of objectors increased and waiting times decreased nationally: an increase from 69.2% to 70.7% among gynecologist objectors; an increase from 56.7% to 59.3% among women who waited less than 14 days to obtain an abortion; and a decrease from 12.4% to 11.1%, among women who waited 22-28 days. A similar “trend” was reportedly found for some regions (e.g., Latium and Piedmont), whereas in other regions (Lombardy and Umbria) the proportion of objectors among gynecologists decreased and the waiting times for abortion increased (the proportion of women who waited less than 14 days decreased and the proportion of women who waited 22-28 days increased). Differently, both the proportion of gynecologist objectors and waiting times for abortion decreased in Emilia-Romagna. The abortion data show different if not opposing “trends” at the regional level: waiting time decreased as the proportion of objectors increased in some regions, while in others waiting time increased as the proportion of objectors decreased (Table 1). In brief, no correlation can be found between the number of objectors and the length of waiting time for abortion. From this perspective, it is not the number of objectors per se that defines access to voluntary abortion, but rather how health trusts organize the delivery of abortion services.

**Table 1:** Proportion of conscientious objectors and women who obtained a voluntary abortion promptly (≤14 days) or later (22-28 days): three comparisons

	Gyn. objectors % (n)			Waiting time ≤ 14 days % (n)			Waiting time 22-28 days % (n)		
Comparison (2006 vs 2009) proposed by the Italian Commitee for Bioethics									
	2006	2009	changes	2006	2009	changes	2006	2009	changes
*Italy*	*69.2 (3780)*	*70.7 (3985)*	*↑*	*56.7 (68217)*	*59.3 (65919)*	*↑*	*12.4 (14875)*	*11.1 (12313)*	↓
Latium	77.7 (443)	80.2 (315)	*↑*	47.8 (7190)	54.0 (7070)	*↑*	17.2 (2584)	13.3 (1738)	↓
Piedmont	62.9 (285)	63.8 (284)	*↑*	51.1 (5635)	60.1 (5705)	*↑*	13.7 (1508)	10.8 (1020)	↓
Lombardy	68.6 (578)	66.9 (560)	↓	58.6 (12763)	56.0 (9868)	↓	11.3 (2463)	11.5 (2024)	*↑*
Umbria	70.2 (73)	63.3 (62)	↓	51.0 (1089)	40.0 (754)	↓	13.3 (284)	19.0 (358)	*↑*
Emilia-Romagna	53.5 (198)	52.4 (205)	↓	56.8 (6510)	62.0 (6712)	*↑*	11.1 (1274)	8.3 (899)	↓
Comparison (2006 vs 2013) proposed by the Italian Ministry of Health									
	2006	2013		2006	2013		2006	2013	
*Italy*	*69.2 (3780)*	*70.0 (3481)*	*↑*	*56.7 (68217)*	*62.3 (61254)*	*↑*	*12.4 (14875)*	*10.2 (10013)*	↓
Latium	77.7 (443)	80.7 (314)	*↑*	47.8 (7190)	54.0 (6020)	*↑*	17.2 (2584)	13.5 (1509)	↓
Piedmont	62.9 (285)	67.4 (269)	*↑*	51.1 (5635)	68.3 (5775)	*↑*	13.7 (1508)	7.4 (626)	↓
Lombardy	68.6 (578)	63.6 (565)	↓	58.6 (12763)	54.4 (8708)	↓	11.3 (2463)	13.5 (2157)	*↑*
Umbria	70.2 (73)	65.6 (63)	↓	51.0 (1089)	43.8 (717)	↓	13.3 (284)	17.6 (288)	*↑*
Emilia-Romagna	53.5 (198)	51.8 (231)	↓	56.8 (6510)	73.7 (6754)	*↑*	11.1 (1274)	4.8 (442)	↓
Comparison (2009 vs 2013) proposed by the authors									
	2009	2013		2009	2013		2009	2013	
*Italy*	*70.7 (3985)*	*70.0 (3481)*	↓	*59.3 (65919)*	*62.3 (61254)*	*↑*	*11.1 (12313)*	*10.2 (10013)*	↓
Latium	80.2 (315)	80.7 (314)	*↑*	54.0 (7070)	54.0 (6020)	≈	13.3 (1738)	13.5 (1509)	*↑*
Piedmont	63.8 (284)	67.4 (269)	*↑*	60.1 (5705)	68.3 (5775)	*↑*	10.8 (1020)	7.4 (626)	↓
Lombardy	66.9 (560)	63.6 (565)	↓	56.0 (9868)	54.4 (8708)	↓	11.5 (2024)	13.5 (2157)	*↑*
Umbria	63.3 (62)	65.6 (63)	*↑*	40.0 (754)	43.8 (717)	*↑*	19.0 (358)	17.6 (288)	↓
Emilia-Romagna	52.4 (205)	51.8 (231)	↓	62.0 (6712)	73.7 (6754)	*↑*	8.3 (899)	4.8 (442)	↓

Data refer to those cited in ‘The no correlation argument’ section of the paper and are an extract of data published in the 2012 opinion of the Italian Committee for Bioethics (2006 vs 2009) and in the 2015 Ministerial report (2006 vs 2013). **↑**: the proportion increased; **↓**: the proportion decreased; **≈**: the proportion did not change

In its 2015 annual report, the Ministry of Health used the same argument based on a comparison between the national data sets for 2006 and 2013 [6]: the proportion of objectors among gynecologists rose from 69.2% to 70%; the proportion of women who waited less than 14 days increased from 56.7% to 62.3%; and the proportion of those who waited 22-28 days decreased from 12.4% to 10.2%. The Health Ministry concluded that while the proportion of objectors had increased, the length of waiting times had decreased – in other words – had improved. As in the previous comparison, the regional data showed a decrease in waiting times despite the increase in the proportion of objectors (e.g., Latium and Piedmont), whereas an inverse “trend” was found for Lombardy and Umbria, and a decrease in both waiting times and the proportion of objectors in Emilia-Romagna (Table 1).

### The limitations of the *no correlation* argument

In authors’ opinion, as currently formulated, the no correlation argument shows significant limitations. First, the way the data were selected limits the extent to which conclusions can be drawn from them. The Italian national surveillance system collects data on voluntary abortion since 1980, and ministerial reports are presented annually to the Italian Parliament. Nonetheless, data collected during two non-consecutive years were compared, assuming that a consistent trend was present in the period between those 2 years and in the previous ones. While any reason to explain this is absent the choice seems to have been an arbitrary one and risks describing random facts related to the years chosen for the comparison as representing a consistent trend.

To demonstrate this limitation, we can apply the procedure used to develop the no correlation argument to data sets from a different couple of years. For example, if we compare data collected in 2009 and 2013, instead of those from 2006 and 2009 (or 2006 and 2013), we will see that at the national level both the proportion of objectors and the length of waiting time for voluntary abortion decreased. Indeed, the proportion of objectors decreased from 70.7% in 2009 to 70% in 2013, the proportion of women who waited 14 days or less to have an abortion increased from 59.3% to 62.3%, while those who waited 22-28 days to have an abortion decreased from 11.1% to 10.2% (Table 1).

Second, the term “correlation” is inappropriately used for the no correlation argument. In everyday life “correlation” means some form of association between events that seem to be linked (e.g., children’s height and age). In statistics, correlation indicates an association between two quantitative variables and it assumes that this association is linear. The degree of this type of association is usually measured by calculating a correlation coefficient (Pearson’s r or Spearman’s rho) on a scale that varies from +1 (perfect positive correlation) to −1 (perfect negative correlation), and 0 means the complete absence of correlation [7]. Based on this definition, what the CNB did is just a direct comparison of prevalences extrapolated from two arbitrarily chosen years.

Third, the no correlation argument excludes a causal effect between conscientious objection and waiting times for abortion based on the lack of a clear association between the proportion of objectors and the proportion of women who obtained an abortion within specific waiting periods (14 days or later). In our opinion, this conclusion is incorrect if not misleading. Correlation studies evaluate whether two events are directly (A increases as B increases) or indirectly (A decreases as B increases) linearly related, but they cannot be used to demonstrate causal effects. Thus, if we take two parameters (A and B) which can assume different values during time and in space (a1, a2,…an e b1, b2,…bn), and we observe that B decreases by a fixed amount for each unit increase in A, we may state that A and B – and the events they describe – are correlated, but we cannot state that B decreases because of an increase in A. Thus, observing an indirect correlation between the proportion of objectors and the proportion of women who obtained a voluntary abortion within 14 days of the request does not mean that the increase in the proportion of objectors caused a decrease in the proportion of women who quickly obtained an abortion. Similarly, finding no correlation (meant as a linear relationship) between two parameters cannot exclude other types of relationships (non linear) between them.

In a previous ecological study in which we used both national and regional data (extrapolated from ministerial reports) from a longer time period (from 1997 to 2011) and took into account the increased workload for non-objectors due to the presence of objectors working at the same facility, we found a correlation (in its statistical meaning) between the gynecologists’ workload and the proportion of women who obtain an abortion within different time intervals. In particular, the increased workload for non-objectors was inversely correlated with the proportion of women who promptly obtained an abortion (within 14 days of their request) and was directly correlated with the proportion of women who obtained an abortion 21 days after the request or even later, at both the national level and in seven Italian regions [8].

Fourth, the *no correlation* argument considers only gynecologist conscientious objectors, whereas conscientious objection to abortion is a very complex phenomenon to describe and understand. It is the result of the interaction of many actors who operate at the same time or at different times in different services: a surgical abortion involves a surgical team composed of a gynecologist, a midwife, and an anesthesiologist, all of which must be non-objectors. Furthermore, these professionals work different shifts and are on duty in different wards (e.g., gynecology, anesthesiology, operating room). It is clear that the proportion of non-objectors among gynecologists and their workload are only two of the many factors that go into the analysis of how conscientious objection may affect access to voluntary abortion.

### Can we state that conscientious objection does not restrict women’s access to voluntary abortion?

The data extrapolated from the ministerial reports are unsuitable to evaluate whether or not conscientious objection restricts access to voluntary abortion among Italian women. Nevertheless, it is not by chance that two recent ECSR judgments stated that the Italian Government did not provide suitable data to prove that the claimants’ thesis were wrong [3, 4]. In order to empirically sustain the thesis that conscientious objection does not restrict women’s access to abortion, other studies are needed that take into account the effect of potential confounders that may influence the relationship between conscientious objection and waiting times for abortion (e.g., age, nationality, civil status, education, previous pregnancies of the women involved, location and opening times of the services, etc.).

A desirable step forward could be to analyze data at the individual level rather than in an aggregate form, as has been done till now. With this approach, we could evaluate for each case of voluntary abortion whether – during her clinical path – the woman, complete with her social, demographic, and clinical characteristics, met objectors or had gone to a health facility that had refused to perform the procedure, how many times, in which facilities, and whether these facts influenced the time interval between the day the abortion was performed and the day it had been requested.

Moreover, it would be important to know how health trusts manage the provision of abortion services: e.g., their number and location (in urban, rural or mountain areas), the number and type of providers (gynecologists, anesthesiologists, midwives, etc.) willing to perform an abortion, the number of surgical voluntary abortions, whether the populations served significantly differ across facilities, waiting times and active and passive mobility for the delivery of voluntary abortion services at each facility. Current abortion legislation does not mandate such analyses, which demand time, dedicated personnel, economical resources, and periodical updates. In addition, such studies work well if supported by central coordination of an adequate number of health trusts in order to obtain a representative sample at the national or regional level.

## Conclusions

The no correlation argument fails to exclude a relationship between the number of conscientious objectors and waiting times for voluntary abortion. Indeed, it cannot exclude the possibility that the increase in the number of objectors restricts women’s access to abortion in Italy. Furthermore, its limitations and methodological flaws underline that the data used till now are insufficient to answer the question. Currently, we do not know the real effect of conscientious objection on access to voluntary abortion in Italy, as compared with other countries where conscientious objection is allowed and involves a relevant proportion of healthcare providers. This is why it is necessary and urgent to conduct further studies designed to better describe the relationship between conscientious objection and waiting times for voluntary abortion. If new data show that the increasing proportion of objectors undermines the efficacy of the legal provisions to ensure the delivery of voluntary abortion, new ways will need to be found to address the conflict between the principles of reproductive autonomy and moral integrity. This would inevitably imply the need to constrain and to better define the terms and conditions for claiming conscientious objection. At that point, nearly 40 years after abortion became legal in Italy, the stage will be set for a more balanced debate on the morality of conscientious objection to abortion and for a re-evaluation of whether it is appropriate to grant this right or it is preferable to follow the example of European countries where conscientious objection is not contemplated.

## Abbraviations

CNB: National Committee for Bioethics
ECSR: European Committee for Social Rights
